# Diagnosis of a Single-Nucleotide Variant in Whole-Exome Sequencing Data for Patients With Inherited Diseases: Machine Learning Study Using Artificial Intelligence Variant Prioritization

**DOI:** 10.2196/37701

**Published:** 2022-09-15

**Authors:** Yu-Shan Huang, Ching Hsu, Yu-Chang Chune, I-Cheng Liao, Hsin Wang, Yi-Lin Lin, Wuh-Liang Hwu, Ni-Chung Lee, Feipei Lai

**Affiliations:** 1 Department of Computer Science and Information Engineering National Taiwan University Taipei City Taiwan; 2 Graduate Institute of Biomedical Electronics and Bioinformatics National Taiwan University Taipei City Taiwan; 3 Department of Medical Genetics National Taiwan University Hospital Taipei City Taiwan; 4 Department of Pediatrics National Taiwan University Hospital Taipei City Taiwan

**Keywords:** next-generation sequencing, genetic variation analysis, machine learning, artificial intelligence, whole-exome sequencing

## Abstract

**Background:**

In recent years, thanks to the rapid development of next-generation sequencing (NGS) technology, an entire human genome can be sequenced in a short period. As a result, NGS technology is now being widely introduced into clinical diagnosis practice, especially for diagnosis of hereditary disorders. Although the exome data of single-nucleotide variant (SNV) can be generated using these approaches, processing the DNA sequence data of a patient requires multiple tools and complex bioinformatics pipelines.

**Objective:**

This study aims to assist physicians to automatically interpret the genetic variation information generated by NGS in a short period. To determine the true causal variants of a patient with genetic disease, currently, physicians often need to view numerous features on every variant manually and search for literature in different databases to understand the effect of genetic variation.

**Methods:**

We constructed a machine learning model for predicting disease-causing variants in exome data. We collected sequencing data from whole-exome sequencing (WES) and gene panel as training set, and then integrated variant annotations from multiple genetic databases for model training. The model built ranked SNVs and output the most possible disease-causing candidates. For model testing, we collected WES data from 108 patients with rare genetic disorders in National Taiwan University Hospital. We applied sequencing data and phenotypic information automatically extracted by a keyword extraction tool from patient’s electronic medical records into our machine learning model.

**Results:**

We succeeded in locating 92.5% (124/134) of the causative variant in the top 10 ranking list among an average of 741 candidate variants per person after filtering. AI Variant Prioritizer was able to assign the target gene to the top rank for around 61.1% (66/108) of the patients, followed by Variant Prioritizer, which assigned it for 44.4% (48/108) of the patients. The cumulative rank result revealed that our AI Variant Prioritizer has the highest accuracy at ranks 1, 5, 10, and 20. It also shows that AI Variant Prioritizer presents better performance than other tools. After adopting the Human Phenotype Ontology (HPO) terms by looking up the databases, the top 10 ranking list can be increased to 93.5% (101/108).

**Conclusions:**

We successfully applied sequencing data from WES and free-text phenotypic information of patient’s disease automatically extracted by the keyword extraction tool for model training and testing. By interpreting our model, we identified which features of variants are important. Besides, we achieved a satisfactory result on finding the target variant in our testing data set. After adopting the HPO terms by looking up the databases, the top 10 ranking list can be increased to 93.5% (101/108). The performance of the model is similar to that of manual analysis, and it has been used to help National Taiwan University Hospital with a genetic diagnosis.

## Introduction

### Background

Modern next-genome sequencing (NGS) technology makes rapid human genome sequencing within a day possible [1,2]. Because of its speed and low cost in comparison with the traditional Sanger sequencing method [3], NGS is being rapidly introduced into clinical and public health laboratory practice, especially for the diagnosis of hereditary disorders.

Although NGS has extremely high throughput and could generate huge amounts of genomic data in a short time, interpreting these data and finding the disease-causing candidates among thousands of variants remain a challenge. To determine the true causal variants of a patient with genetic disease, physicians often need to view numerous features on every variant manually and search for literature in different databases to understand the effect of a genetic variation. Another challenge is in finding the genetic variants that have a strong correlation with patient’s phenotype. Physicians often select useful keywords from patient’s electronic medical records (EMRs) manually to search for articles in several genetic databases such as Online Mendelian Inheritance in Man (OMIM) [4] and GeneReviews [5] to decide whether a variant is correlated with a genetic disease. It is thus a burden for physicians to go through these laborious and time-consuming processes case-by-case, especially when the number of inherited disease–associated germline mutations published per year has increased exponentially in the last decade [6].

Nowadays, many studies use machine learning methods to solve numerous problems in genomics and genetics. The field of machine learning promises to enable computers to assist humans in making sense of large, complex data sets. After variant annotation, there is a variant list with hundreds of columns that humans are not capable of interpreting one-by-one. As machine learning significantly surpasses human-level performance, especially with structured data, we consider using a machine learning method to analyze variants from NGS and find the target gene.

To address these problems, it is important and necessary to have a high-performance method to filter candidate variants from NGS results and immediately find target variants related to a patient’s disease. Recently, many tools such as Exomiser [7], DeepPVP [8], Xrare [9], VarSight [10], Phenolyzer [11], Fabric GEM [12], MOON [2], CADD [13], and MetaSVM [14] have been developed to identify potentially causative variants that are relevant to patient’s phenotype in rare disease diagnosis. Exomiser integrates information including calculated gene-specific phenotype score, variant allele frequency (Multimedia Appendix 1), and predicted pathogenicity of several alleles to prioritize disease-causative variants/interactions. Fabric GEM utilizes Bayes factor to prioritize variants with the support of a gene-phenotype score calculated by Phevor [15] and variant prioritization result of several tools including ANNOVAR, VAAST, and Phen-Gen. MOON integrates the result of annotation of several variants and prioritization tools to achieve variant prioritization using several kinds of machine learning models. Gene-phenotype scores calculated by Phevor using Human Phenotype Ontology (HPO) terms extracted from electronic health records (EHRs) of patients are also considered by MOON. CADD utilizes logistic regression to integrate information including context of surrounding sequence, biological constraints, epigenetic measurements, and result of several variant annotation tools to build a predictive model for variant deleteriousness. MetaSVM [14] gathers result of 9 deleteriousness prediction scores including PolyPhen-2 [16], SIFT [17], MutationTaster [18] to build a support vector machine (SVM) deleteriousness predictive model. Although these tools adopt different approaches, including logistic regression and deep neural networks, to prioritize variants, most can only recognize the phenotypes defined in the HPO term [19]. In this work, we developed the AI Variant Prioritizer module based on a machine learning approach that can output the rank of single-nucleotide variants (SNVs) and small insertions/deletions (indels) from whole-exome sequencing (WES) data with the input of free-text phenotypic description or EHR.

In this research, we aimed to implement a website, AI Variant Prioritizer, that uses data from NGS bioinformatics pipelines with machine learning to make a prediction about the most possible disease-causing variants among SNVs and patient’s phenotype. The data generated from NGS pipelines are all structured with annotations from several tools including ANNOVAR, Nirvana, Variant Effect Predictor (VEP), and InterVar and additional information from multiple databases queried by MViewer (Mutation Viewer) [20]. To simplify the interpretation process, we integrate the keyword extraction tool to generate the phenotype from EMRs automatically. Our system takes candidate variants filtered by MViewer and patient’s EMRs as its input and outputs a list of SNVs with rank and probability of being disease causing. Instead of checking every variant manually, this system can assist researchers and physicians in focusing on those with higher disease-causing probability and save a lot of time. Moreover, we implement a web application programming interface (API) for our system so that the ranking function could be integrated into MViewer. Thus, physicians are able to interpret the results of genetic variation with a single application instead of adopting numerous services.

### Data Description

In our research, we focus on patients who have been diagnosed with rare Mendelian diseases. Our data are collected mainly from the rapid exome project of Department of Medical Genetics, National Taiwan University Hospital (NTUH). To build the model with more data, we also applied for several WES data that are deposited in the dbGaP database (project ID 20911). The data we use are the dbGaP accession phs000711.v5.p1 by Baylor Hopkins Center for Mendelian Genomics.

The conditions under which we collect patients’ sequencing data to meet the requirements of this research are as follows:

- Patients who were diagnosed with genetic disorders.
- Patients who received WES or targeted panel sequencing and diagnosed with at least one disease-causing variant.
- Patients whose phenotype information is available.

Our data from NTUH include patient demographics, variant call format (VCF) file output by the NGS bioinformatics pipeline, and phenotype information from electrical medical records. Data from dbGaP also include patient demographics, VCF file, and clinical conditions. All data are deidentified and will not invade patients’ privacy. We include sex in patient demographic information as a feature in our model because some human genetic disorders are sex linked. Sex-linked diseases are caused by mutations in genes on X or Y chromosomes and passed down through families.

### Variant Call Format File

As the end product of the NGS bioinformatics pipeline, the VCF is a generic format for storing DNA polymorphism data such as SNPs, insertions, deletions, and structural variants. The format was developed for the 1000 Genomes Project and has also been widely adopted by other projects. Every VCF file consists of 2 two parts: header section and data section. The header contains metadata about the tags and annotations in the data part. It can be also used to provide information related to the history of the data and file. The last line in the header contains the column headings for the data part. The data section is tab separated into 9 columns and reports a mutation for each row. Columns include CHROM, POS, ID, REF, ALT, QUAL, FILTER, INFO, and FORMAT.

### Phenotype Information

For the data from NTUH, we extract patient’s phenotypic information from clinicians’ history summary. It mainly records a brief summary of patient’s illness, clinical diagnosis, and the reason(s) why each patient was admitted. We also collect the phenotype keywords provided by doctors based on the symptom of each patient for model validation. For the data from dbGaP, because EHRs are not available, we will use the clinical condition of the patient instead. For the clinical condition that can be found in OMIM databases, we will extract the corresponding description of phenotypes as the phenotypic information to be used in our research.

## Methods

### Workflow

#### Overview

**Figure 1:**
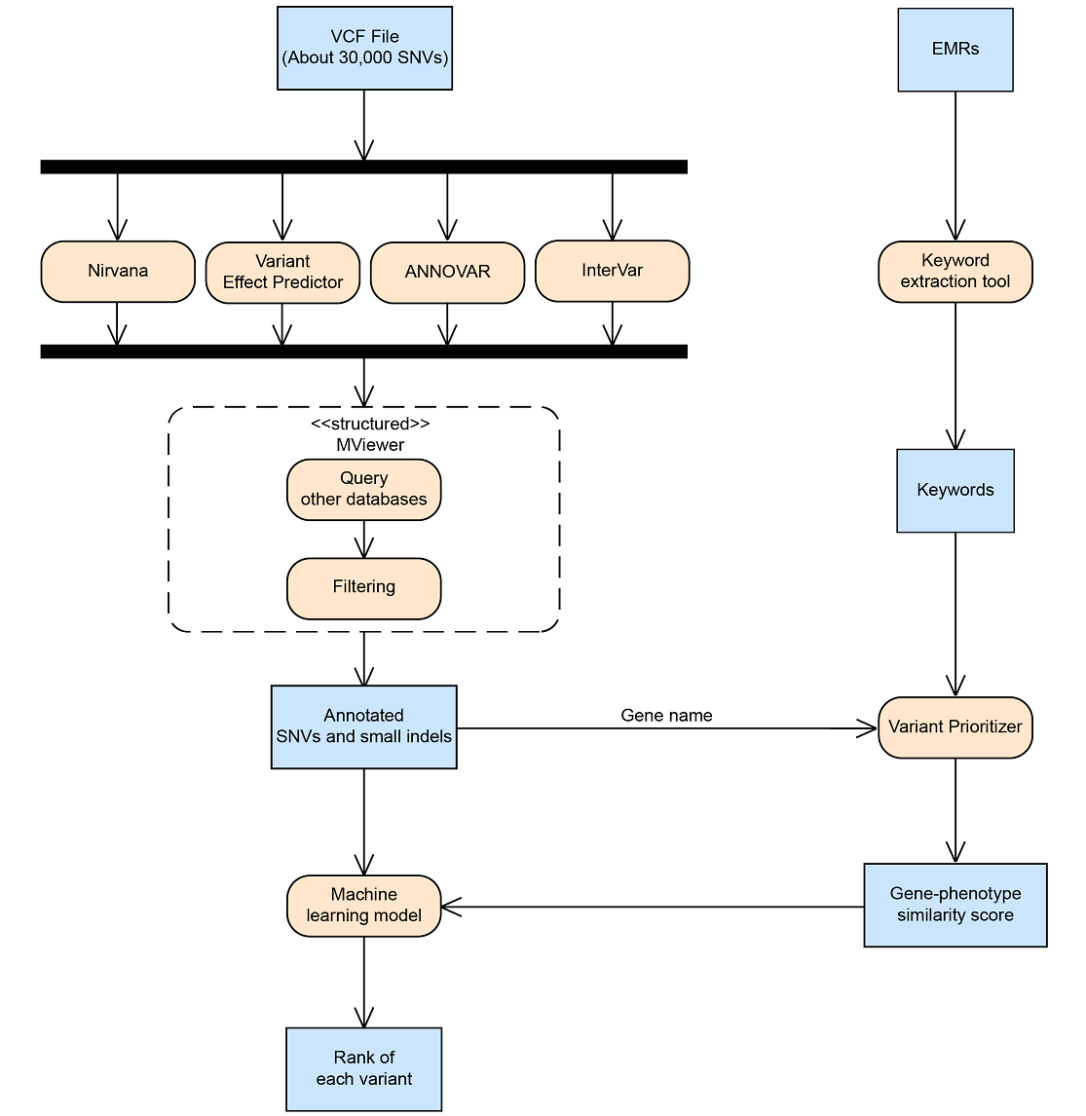
The workflow of research. EMR: electronic medical record; indel: insertion/deletion; MViewer: Mutation Viewer; SNV: single-nucleotide variant; VCF: variant call format.

Figure 1 shows the workflow of our research. We collected VCF of each patient from WES and panel sequencing and then annotated the variants using several tools. After variant annotation, we used our in-house software (MViewer [20]) to query additional external databases and filter for candidate variants. We then used the gene name of these candidate variants and keywords extracted by keyword extraction tools from EMRs to query Variant Prioritizer [21]. The gene similarity scores generated by Variant Prioritizer and columns of annotated variants were used as features to train a machine learning model. This model ranks each variant that represents its disease-causing probability. We will demonstrate the details of each step in the following sections.

#### Variant Annotation

We collected each patient’s NGS sequencing data in the VCF file and got annotations from several tools, including ANNOVAR [22], VEP [23], Nirvana [24], and InterVar [25]. For additional information that the aforementioned tools will not provide, we used software to import some public data sources, including ClinVar [26], Human Genome Mutation Database (HGMD) [27], and Taiwan Biobank [28]. A detailed description of these annotation fields is summarized in Textbox 1.

**Description of annotation fields.**

**Allele Frequency**

This describes the fraction of gene copies of a particular allele in a defined population. Allele frequency is calculated by dividing the number of copies of a particular allele in a population by the total number of all alleles for that gene in a population. It refers to how common an allele is in a population.

**Functional Prediction Score**

A range of scoring algorithms with capability to predict the potential deleteriousness of variants based on different information in them, such as their sequence homology, protein structure, and evolutionary conservation. These scoring methods include function prediction scores, conservation scores, and ensemble scores.

**Pathogenicity**

Clinical significance variants reported in 2 public databases, ClinVar and Human Gene Mutation Database (HGMD), that store information on gene mutation(s) related to human-inherited disease. Both classify variants as disease causing or disease associated by manual curation.

**Clinical Interpretation**

The American College of Medical Genetics and Genomics (ACMG) and the Association for Molecular Pathology (AMP) published standards and guidelines for the clinical interpretation of sequence variants with respect to human diseases on the basis of 28 criteria [29]. These criteria are as follows: the criteria (16 overall) for classifying variants as pathogenic or likely pathogenic are very strong (PVS1), strong (PS1-PS4), moderate (PM1-PM6), or supporting (PP1-PP5), whereas the criteria (12 overall) for classifying variants as benign or likely benign are standalone (BA1), strong (BS1-BS4), or supporting (BP1-BP7).

**Gene-Level Constraint**

Constraint on gene expression levels has been shown to influence patterns of genetic variation within humans [30]. For example, some genes are unusually depleted for loss of function and are thought to be constraint with respect to their expression. The Genome Aggregation Database (gnomAD) provides predicted constraint metrics track set that contains metrics of pathogenicity per gene as predicted and identifies genes subject to strong selection against various classes of mutation. These include several subtracks of constraint metrics calculated at gene, transcript, and transcript region levels.

**Disease Inheritance**

Patterns of inheritance that a trait or disorder associated with a variant can be passed down through families, such as autosomal dominant, autosomal recessive, X-linked, and mitochondrial inheritance. We used the patterns defined in OMIM (Online Mendelian Inheritance in Man) as our data.

**Others**

Additional information about genetic variants such as the gene name, genotype, and the functional consequence on the different transcripts for a gene or in proximal regulatory regions.

#### Variant Filtering

There are on average 40,000 variants per proband in WES data. However, most of them are benign and not related to the symptoms. Only a small number of these variants are likely to be deleterious or relevant to the patient’s disease. In a standard clinical analysis process, physicians only focus on variants that might be pathogenic or unknown. As our model aims to assist researchers and physicians with their clinical exome reading, reducing the number of variants and focusing on the variants that are more likely to be responsible for the disease are necessary.

For the purpose of generating candidate variants, we used the filter provided by MViewer to remove the variants that are not likely to be deleterious. The filters and criteria are listed in Table 1. For filters that contain more than 1 column, if a variant meets any of their criterion, it will remain in the data. We got approximately 700 SNVs per patient after variant filtering.

**Table 1:** Filter criteria.

Filter	Column	Criteria
Max allele frequency	Max Allele Frequency	≤0.01 (include no data)
Nonsynonymous missense mutation	ExonicFunc.refgene	“nonsynonymous”
Stop gain	Consequence ExonicFunc.refgene	“stop_gained” “stopgain”
Splice	Consequence Func.refgene	“splice_region_variant” “splice_acceptor_variant” “splice_donor_variant” “splicing”
Frameshift	Consequence ExonicFunc.refgene	“frameshift_variant” “feature_truncation” “feature_elongation” “frameshift”
Initial codon	Consequence	“start_lost”
Deletion	Type Consequence ExonicFunc.refgene	“deletion”
Insertion	Type Consequence ExonicFunc.refgene	“insertion”
Inframe deletion	Consequence ExonicFunc.refgene	“inframe_deletion” “nonframeshift deletion”
Exon/splice site	Func.refgene Consequence	“exonic” “splicing” “coding_sequence_variant” “frameshift_variant” “incomplete_terminal_codon_variant” “inframe_deletion” “inframe_insertion” “missense_variant” “splice_acceptor_variant” “splice_donor_variant” “splice_region_variant”

#### Phenotype Extraction

##### Overview

The phenotype information used in this research is from clinicians’ history summary. The records were all free text and the length of texts varied from less than 10 to more than 300 words. In the clinical analysis process, it is time consuming for physicians to go through the medical records and identify the phenotype keywords manually. To solve this problem, we used several keyword extraction tools to automatically generate keywords related to phenotype from free-text medical records. The keyword extraction tools applied in our research are listed in the following sections.

##### MetaMap

MetaMap [31] is a widely used application providing access to the concepts in the Unified Medical Language System (UMLS) Metathesaurus [32]. The UMLS Metathesaurus is a compilation of names, relationships, and associated information from a variety of biomedical naming systems representing different views of biomedical practice or research. It comprises over 1 million biomedical concepts and 5 million concept names [33]. MetaMap is able to map every word in the texts to UMLS concepts, but we just wanted to focus on those associated with phenotypes and diseases. Thus, we extracted the words that are classified as the semantic types of the following: (1) injury or poisoning, (2) cell or molecular dysfunction, (3) genetic function, (4) disease or syndrome, (5) sign or symptom, (6) tissue.

##### Doc2Hpo

Doc2Hpo [34] is a web application using natural language processing (NLP) techniques to parse clinical note and get the phenotype concept curation as the HPO term. There is a parsing engine that will automatically recognize the phenotype concepts from the input. Doc2Hpo applies an algorithm called NegBio for negation detection in the input data. After that, there are several NLP engines responsible for HPO concept extraction. We used 3 of these engines and compared the performance of each of them. The first NLP engine is a string-based method that leverages the algorithm for concept extraction. The second engine is the online NCBO Annotator [35] API for HPO concept recognition. The last engine we adopt is MetaMap Lite, which is a fast version of MetaMap that provides near–real-time named entity recognition. The MetaMap Lite engine first identifies clinical terms in the texts and maps them to standard UMLS concepts. The UMLS concepts will then be further mapped to HPO concepts. Results generated by Doc2Hpo are HPO terms, whereas keywords extracted by MetaMap are nonHPO terms.

### Phenotype-Gene Similarity Score

Another method to construct the connections between genes and keywords is using the Okapi BM25 ranking function. Okapi BM25 is usually used by search engines, such as Google and Bing, to rank matching documents according to their relevance to a given search. One of the most prominent instantiations of the function is as the following equation:



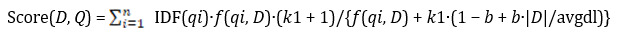



where score(*D*, *Q*) represents the Okapi BM25 score of a document *D* when given a query Q, containing keywords *q*1, *q*2,...,*qn*; *f*(*qi*, *D*) is *qi*’s term frequency in the document *D*; |*D*| is the length of document *D* in words; avgdl is the average document length among all documents; *k*1 and *b* are constants (=1.2 and 0.8, respectively); and IDF(*qi*) is the inverse document frequency (IDF) weight of the query term *qi* and is usually defined as:


IDF(*qi*) = ln [(*N* – *n*(*qi*) + 0.5]/[*n*(*qi*) + 0.5 + 1]

where *N* is the number of documents and *n* is the number containing the keywords.

In this research, we propose an idea using gene description from OMIM and GeneReviews as documents and keywords as query to implement the Okapi BM25 ranking function. Therefore, we can use the Okapi BM25 score to represent the relationship between gene description and keywords. The higher score that gene description gets from keywords indicates stronger connection between that gene and keywords. Rank values were based on the Okapi BM25 ranking function mentioned before with some other parameters. Compared with the Okapi BM25 regular formula, rank value replaces the IDF function with Robertson-Spärck-Jones weight [36]. The IDF function is a measure of how much information the word provides, that is, whether the word is common or rare across all documents. For example, the term “the” is very common in every document, so term frequency will be inclined to falsely highlight the documents that happen to use the word “the” more frequently. Hence, the IDF function is dedicated to reducing the weight of words that appear very frequently among all documents. In contrast to the regular IDF function, the Robertson-Spärck-Jones weight adds relevant parameters of documents and increases the precision of rank score.

We get the phenotype-gene similarity score of each SNV from Variant Prioritizer, a text mining tool that outputs the rank and score of genes by entering symptoms as keywords. Variant Prioritizer uses the Okapi BM25 ranking function [37] to construct the connections between genes and keywords. Gene descriptions from OMIM, GeneReviews, Entrez Gene [38], and PubTator [39] serve as data sources and keywords as query to implement the Okapi BM25 score using the full-text search method. It returns a column called RANK that includes ordinal value from 0 to 1000. The RANK score is based on the following formula:



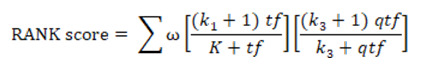



where ω is the Robertson-Spärck-Jones weight [36], which is defined as ω = log [(*r* + 0.5)∙(*N* – *n* – *R* + *r* + 0.5)]/[(*R* – *r* + 0.5)∙(*n* – *r* + 0.5)], in which *R* is the number of known relevant documents and *r* is the number of these containing the term; *tf* is the frequency of the word in the property queried within an article; *qtf* is the frequency of the term in the query; and *K* is defined as follows:

*K* = *k*_1_[(1 – *b*) + *b*(*dl*/avgdl)]

where *dl* is the property length, in word occurrence; avgdl is the average length of the property being queried, in word occurrence; and *k*_1_, *b*, and *k*_3_ are constants (=1.2, 0.75, and 8.0, respectively).

We employed the Variant Prioritizer API to get the RANK value from each data source as gene similarity score to represent the association between each SNVs and extracted keywords. We kept the maximum and minimum scores of rank values (4 overall) as 2 separate features for model building.

### Ethical Considerations

This retrospective cohort study was approved by the Institutional Review Board (IRB) of the National Taiwan University Hospital (IRB number: 201710066RINB). We confirm that all experiments were performed in accordance with relevant guidelines and regulations. The data retrieved from EHRs were deidentified and could not be linked to the patients’ identity by the research team. The need for written informed consent was waived and confirmed by the National Taiwan University Hospital IRB (201710066RINB) because this was a retrospective cohort study with deidentified data. This regulation complies to Health Insurance Portability and Accountability Act (HIPAA) that there are no restrictions on the use or disclosure of deidentified health information.

### Data Preprocessing

#### Overview of Steps

After variant annotation of the VCF file, we preprocessed our data into a model-acceptable format. Data preprocessing is an extremely important step in machine learning because the quality of data can directly affect the ability of a model to learn. It includes various operations and each operation aims to help machine learning build better predictive models. The data preprocessing operations used in this research are explained in the following sections.

#### Missing Value Handling

In real world, the data usually have missing values. AsFor example, in the genotype variable most machine learning methods cannot deal with null value, it is pivotal to identify and correctly handle the missing values. Basically, the missing values can be handled using various techniques such as deletion or imputation [40]. Deletion removes all data for an observation that has 1 or more missing values. However, if there are many columns with missing values, then deletion will result in the lack of data. Therefore, for some columns we used imputation by substituting the missing values in our data set with mean and for some columns we just simply replaced the missing value with a valid value such as 0.

#### One Hot Encoding

Many machine learning algorithms cannot operate on categorical data directly. They require all input features to be numeric. Basically, categorical data contain label values rather than numeric values. As a consequence, categorical data must be converted into a numerical form so that they can be used in the machine leaning model. One hot encoding is a widespread approach for dealing with categorical data. One hot encoding transforms a categorical column to a multidimensional vector. It creates new columns, indicating the presence of each possible value from the original data.

For example, in the genotype variable, there are 3 categories: homozygous (hom), heterozygous (het), and hemizygous (hem). Therefore, 3 binary variables [hom, het, hem] are needed. If genotype of a variant is heterozygous, we use [0,1,0] to represent it.

#### Data Normalization

For continuous data, there are a few with different ranges. If we apply features in very different ranges to some machine learning models such as logistic regression, the feature with broader range will intrinsically influence the result more owing to its larger value. However, this does not necessarily mean that this feature is more important as a predictor. Therefore, we used normalization techniques as a solution to overcome this problem. Normalization is the rescaling of the data from the original range so that all values are within the range of 0 and 1. We rescale all continuous values by min-max normalization. The general formula is as follows:

*X*norm = (*X – X*min)*/*(*X*max *– X*min)

where *X* is the original value and *X*norm is the normalized value. This will make the maximal value map to 1 and the minimal value map to 0. In addition to the aforesaid data preprocessing techniques, we handled different data types in different ways and created some new features for model building. In the following sections, we elaborate on each data type preprocessing and combine them in the end.

#### Functional Prediction Score

Functional prediction scores including SIFT [17], PolyPhen2 HDIV [16], PolyPhen2 HVAR [16], LRT [41], MutationTaster [18], MutationAssessor [42], FATHMM [43], PROVEAN [44], MetaSVM [14], MetaLR [14], M-CAP [45], CADD [13], GERP++ [46], DANN [47], fathmm-MKL [48], GenoCanyon [49], fitCons [50], PhyloP [51], PhastCons [52], and SiPhy [53] were from ANNOVAR. We used converted rank scores provided by ANNOVAR instead of the original score because all these scores are always within the range of 0 and 1. Besides, converted rank scores from different algorithms are monotonic in the same direction. That is, a higher score indicates that the variant is more likely to be damaging [54]. For splice site prediction, we imported the MaxEntScan score using the VEP plugin. We defined a new column called MaxEntScan significance. The value is 1 when the value of MaxEntScan alt is smaller than 3 and MaxEntScan variation is smaller than 30%; otherwise the value is 0. We used clinical significance reported in ClinVar and computed rank score from the HGMD. The HGMD computed rank score is a probability of pathogenicity between 0 and 1, with 1 being most likely disease causing compared with other HGMD entries.

#### Clinical Interpretation

We employed clinical interpretation of each genetic variant based on the American College of Medical Genetics and Genomics/Association for Molecular Pathology (ACMG/AMP) 2015 guideline, which is generated by InterVar. We calculated the ACMG score developed by Xrare to represent its overall pathogenicity. The ACMG score is a weighted sum score based on multiple evidence (n=14) with the following weights for each term: PVS1:6, PS1:4, PM1:2, PM2:2, PM4:2, PM5:2, PP2:1, PP3:1, BA1:9, BS1:3, BS2:3, BP3:1, BP4:1, BP7:2 [9]. We collected gene-level constraint features including pLI, pRec, syn_z, and mis_z from the Genome Aggregation Database (gnomAD). We used the patterns of inheritance defined in OMIM as our data. For variants that contain multiple patterns, we calculated the occurrences of each pattern and stored it as a feature. We also get some additional information about each variant from ANNOVAR such as genotype, regions that a variant hits, and read depths. The quality of each variant is also collected from the VCF file. As the genotype annotated by ANNOVAR does not contain hemizygous alleles, we replaced the genotype feature of all male patients’ chromosome X with hemizygous alleles. In addition, we collected functional consequence on the different transcripts for a gene or in proximal regulatory regions using Nirvana.

#### Labels

The goal of our research was to identify the disease-causing variants with SNVs (ie, we classify a variant as disease causing or not). As machine learning algorithms learn how to assign a class label to a test case from examples, it is necessary to assign a class label to all input training sets. We used the 0/1 label to represent whether a variant is disease causing or not. If a variant is causative, we assigned label 1 to it; otherwise the label is 0. Details about all the features used in our model are presented in Multimedia Appendix 2.

### Feature Selection

After data preprocessing, we got 94 features for each variant. To reduce the high dimension of the input data set while retaining the discriminatory information for classification problems, we applied univariate feature selection techniques from scikit-learn [55] packages to identify the relevant variables in a data set and eliminate the useless variables. Feature selection helps to reduce the noise in the data set and lets the model focus on the relevant signals.

There are several scoring functions provided by scikit-learn univariate feature selection modules. We used mutual information classifier to select the most relevant variables. Mutual information [56] between 2 random variables is a nonnegative value, which measures the general dependence of variables without making any assumptions about the nature of their underlying relationships [57]. The mutual information between 2 discrete random variables X and Y is defined as follows:



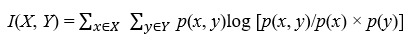



where *p*(*x*, *y*) is the joint probability density function of *X* and *Y*, and *p*(*x*) and *p*(*y*) are the marginal density function. The mutual information determines the similarity between the joint distribution *p*(*x*, *y*) and the products of the factored marginal distributions. The larger the value means the greater the relationship between the 2 variables. The calculated value is equal to 0 if and only if the 2 variables are independent.

We performed the feature selection process using only the training set to determine the relevant variable. Further, the number of features we selected is based on model evaluation with 10fold cross validation

### Building Model

To construct a model by machine learning algorithm, we split the data into 2 groups. As our model aims to assist physicians with their clinical exome data interpretation process, the exome data from the dbGaP database and the targeted gene panel sequencing data from NTUH were set as training set, and the WES data from NTUH were set as testing data. which can only be used on model evaluation. The external validation set consisted of 90 most recent NTUH WES data, which help to make sure that our model can make predictions in future clinical use. Details about the training and testing sets are listed in Table 2.

To build the machine learning model, we implemented the random forests algorithm [58] provided by scikitlearn packages. The selection of hyperparameters is based on a grid search with 10fold cross validation. Random forest was first proposed by Leo Breiman in 2001 [58]. It is an ensemble classifier that evolves from decision trees. Actually, random forests are a combination of decision trees such that each tree depends on the values of a random vector sampled independently, with the same distribution for all trees in the forest [59]. A forest of trees is grown as follows:

- The training set is a bootstrap sample from the original training set.
- The number of trees to build and the number of variables randomly sampled as candidates at each split m-try are set by the user, where m-try is less than the total number of variables.
- At each node, m-try variables are selected at random, and the node is split on the best split point among m-try. This process iterates until the tree grows to its maximal depth.
- For test case prediction, as a test vector **x** is put down at each tree, it is assigned the average of **y** values at the node it stops at. The average of these overall trees in the forest is the predicted value for **x**. The predicted value for classification is the class getting the plurality of the forest votes..

The function we used to measure the quality of a split is Gini impurity. Gini impurity is the probability of incorrectly classifying a randomly chosen element in the data set if it were randomly labeled according to the class distribution in the data set [60]. In decision tree learning it is defined as 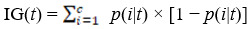, where *c* is the number of classes and *p*(*i*|*t*) is the probability of randomly picking an object of class *i* at node *t*. The optimal split from a root node when training a decision tree is chosen by maximizing the Gini gain, which is calculated by subtracting the weighted impurities of the branches from the original impurity.

**Table 2:** The training, testing, and external validation sets used in this study.

Data	Training set	Testing set	External validation set
Source	dbGaP^a^, NTUH^b^ panel	NTUH WES^c^	New NTUH WES
Patients, n	381	108	90
Filtered variants, n	125,693	80,083	109,857
Causative variants, n	478	134	100

^a^dbGaP: Database of Genotypes and Phenotypes.

^b^NTUH: National Taiwan University Hospital.

^c^WES: whole-exome sequencing.

### Performance Evaluation

To evaluate our model performance of true causative variant prioritization, we used the ranking statistics mentioned in VarSight. After we applied the binary classification process to all variants, we got a probability for each variant that represents the probability of this variant to be disease causing. We ranked the variants for each patient from the highest to lowest probability and quantified the percentage of the target variants that were ranked in the top 1, 5, 10, 20.

## Results

### Feature Selection

**Figure 2:**
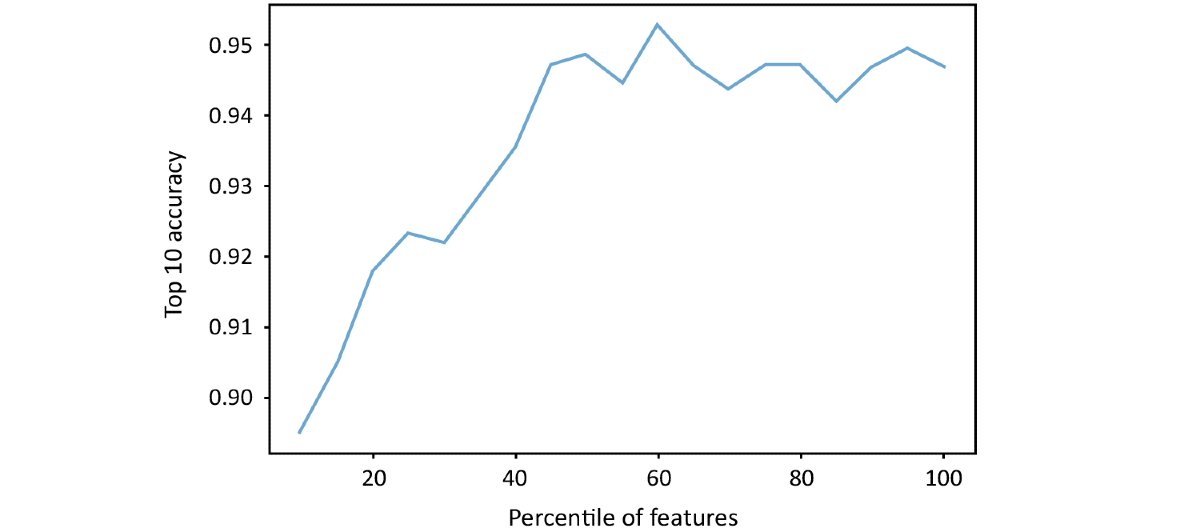
The top 10 accuracy on 10-fold cross validation using different percentage of features.

For the feature selection, we used univariate feature selection based on the SelectPercentile method in scikitlearn package. The classifier we chose is the mutual information classifier. Only the training set was used for selecting the most relevant features. Further, we applied 10fold cross validation to decide the number of features for model training. In Figure 2, we present the top 10 accuracy on 10fold cross validation using different percentages of features. As using 60% of features achieves the highest accuracy, 56 features (60% of total 94 features) with the highest estimated mutual information were selected for the final model building.

### Model Performance

We evaluated the model with our testing set. As mentioned in Table 2, the testing set comprised 108 patients who received WES with at least one disease-causing variant diagnosed by doctors. Multimedia Appendix 3 presents detailed information about their causative variants, keywords, and the corresponding HPO term. The keywords and HPO term are determined by doctors based on the phenotype of each patient.

### Prediction With Different Keyword Extraction Tools

**Figure 3:**
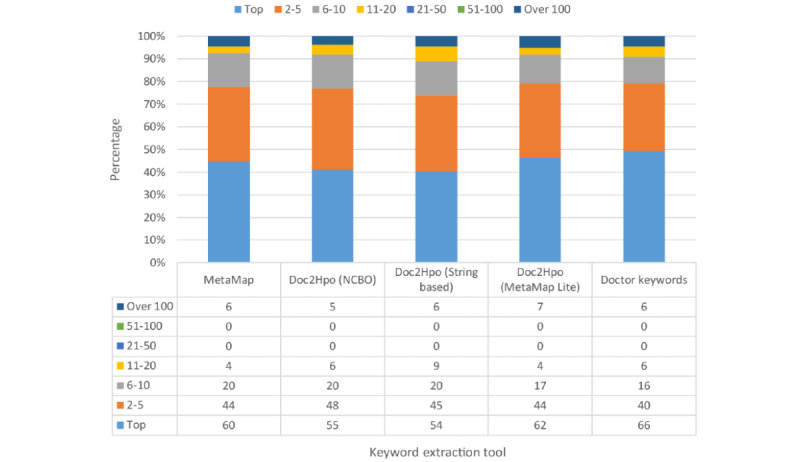
Percentage distribution of ranks.

**Figure 4:**
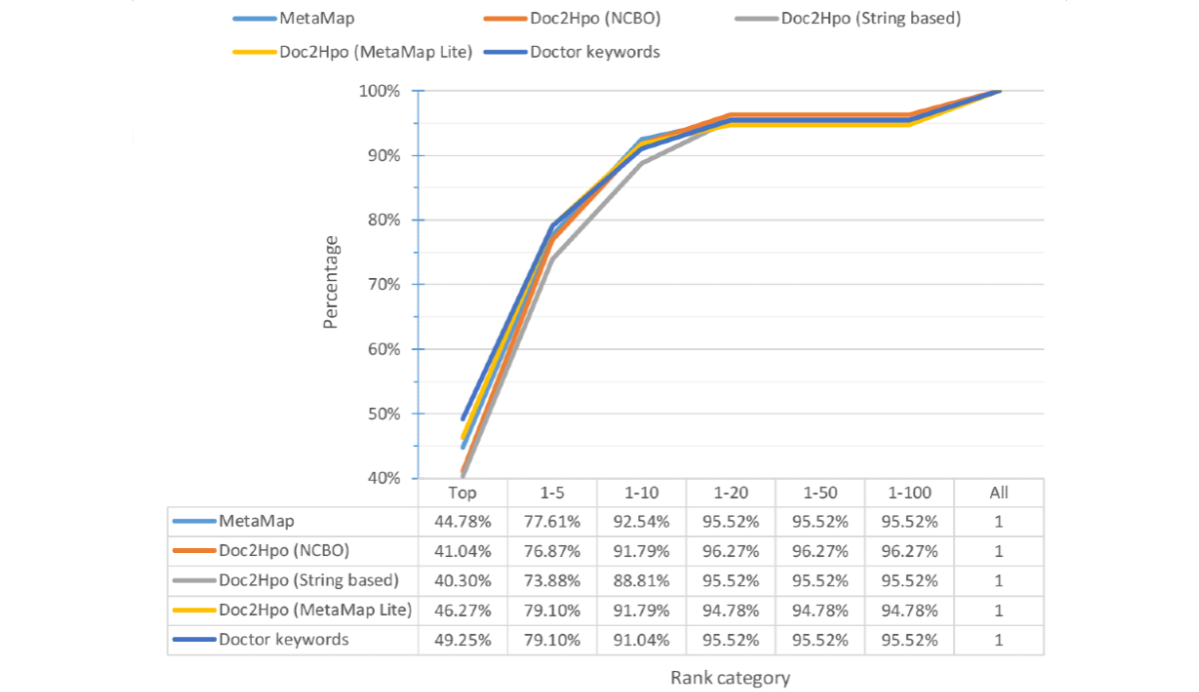
Cumulative percentage distribution of ranks. NCBO: National Center for Biomedical Ontology.

Figure 3 shows the percentage distribution of the ranking of target variants and Figure 4 shows the cumulative rank result of models using different keyword extraction tools. When using tools to extract phenotypes from abstracts, our model can assign the target variants to the top rank for over 40% (60/134, 44.8%) of the total variants. The top 10 accuracies of models are around 90% (124/134, 92.5%), irrespective of the keyword extraction tool used. Compared with the keywords provided by professional doctors, applying tools to extract keywords had lower top 1 accuracy but comparable top 10 accuracy. This indicated that in most cases our model can successfully rank the true causative variants in the front of the variant lists and the rank is slightly influenced by the input keywords.

We built a random forest model based on the method described in the previous section and evaluated it with our testing set based on different keyword extraction tools. We succeeded in locating 92.5% (124/134) of the causative variant in the top 10 ranking list among an average of 741 candidate variants per person after filtering. The performance of the model is similar to that of manual analysis, and it has been used to help National Taiwan University Hospital with a genetic diagnosis.

Figures 3 and 4 show the percentage distribution of the ranking of target variants and the cumulative rank result of models using different keyword extraction tools, respectively. When using tools to extract phenotypes from abstracts, our model can assign the target variants to the top rank for over 40% (60/134, 44.8%) of the total variants. The top 10 accuracies of models are around 90% (124/134, 92.5%), irrespective of the keyword extraction tool used. Compared with the keywords provided by professional doctors, applying tools to extract keywords has lower top 1 accuracy but comparable top 10 accuracy. It represents that in most cases our model can successfully rank the true causative variants in the front of the variant lists and the rank is slightly influenced by the input keywords.

### Other Machine Learning Methods

**Figure 5:**
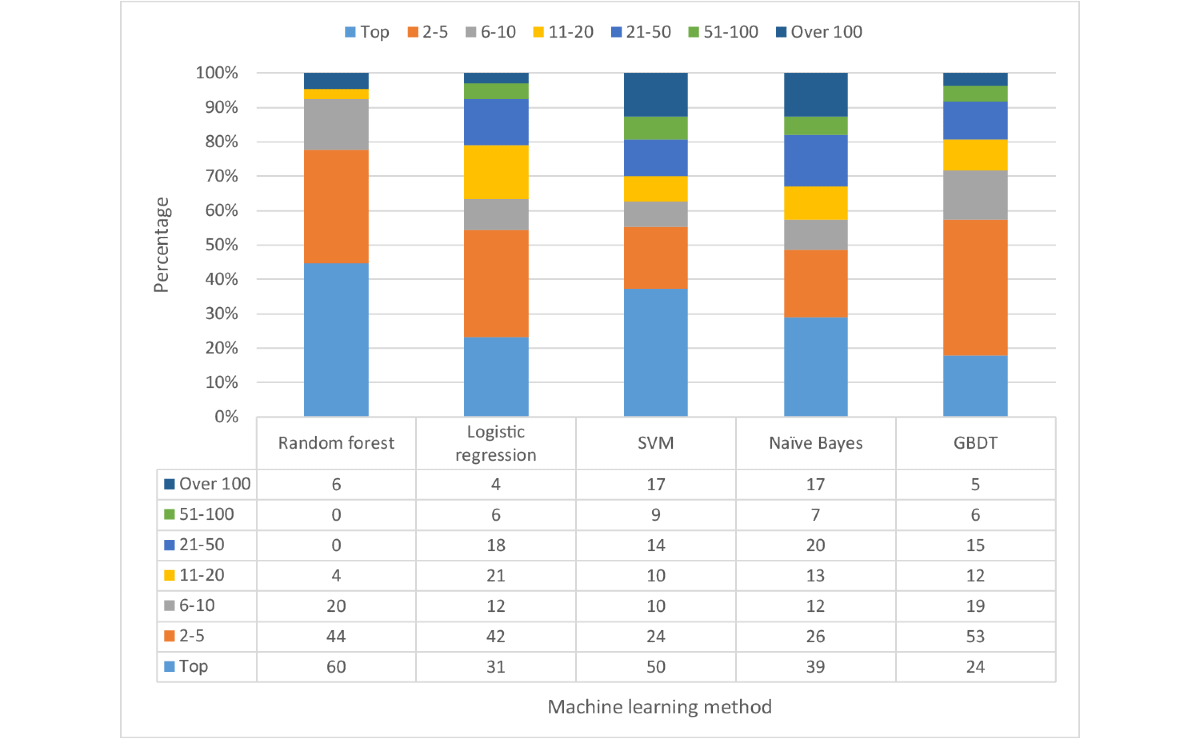
Percentage distribution of ranks. GBDT: gradient boosting decision tree; SVM: support vector machine.

**Figure 6:**
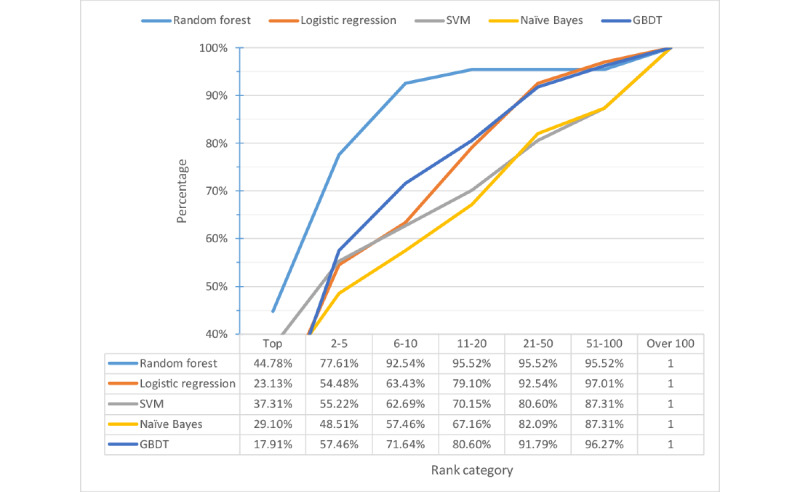
Cumulative percentage distribution of ranks. GBDT: gradient boosting decision tree; SVM: support vector machine.

We also evaluated other machine learning methods and compared their performance with random forest. These methods include logistic regression, Gaussian naive Bayes, SVM with RBF kernel, and gradient boosted decision trees. The selection of hyperparameters for each algorithm was based on grid search with 10-fold cross validation. We used MetaMap as the keyword extraction tool and our testing data to test the performance of each method. The percentage distribution of the ranking of target variants by each machine learning method and the cumulative rank result of each model are shown in Figures 5 and 6, respectively. As random forest got the highest top 10 accuracy, we finally chose random forest as our machine learning algorithm.

## Discussion

### Principal Findings

We have implemented a website, AI Variant Prioritizer, which uses data from NGS bioinformatics pipelines with machine learning to make a prediction about most possible disease-causing variants among SNVs and patient’s phenotype data. This system can assist researchers and physicians by focusing on those with higher disease-causing probability and reducing the average turnaround time (by 1 day) of the entire WES pipeline, from DNA extraction to clinical diagnosis. Moreover, we have implemented a web API for our system so that the ranking function could be integrated into MViewer. Thus, physicians can interpret the results of genetic variation with a single application instead of adopting numerous services.

For comparison, we used our testing data to run several prioritization tools including AMELIE [61], VarElect [62], Exomiser, Phenolyzer, and Variant Prioritizer. As AMELIE and Exomiser can only accept phenotypes defined in HPO terms, we entered HPO terms determined by doctors as their input. Phenolyzer can identify both disease terms and HPO terms. However, if the terms do not match in their databases, it will not return any record. Hence, we also used HPO terms as input for Phenolyzer. VarElect, Variant Prioritizer, and our model can identify free-text descriptions. Therefore, we imputed the keywords provided by doctors as input for testing. AMELIE, VarElect, and Variant Prioritizer only prioritize the gene list instead of the variant list. Hence, we evaluated the result for gene-based prioritization instead of variant-based prioritization. That is, for each patient, if the tools prioritize target gene in the top 1, 5, 10, 20, 50, and 100 of our filtered gene lists, this patient will be counted. All the tools use the default settings provided in their websites to run.

**Figure 7:**
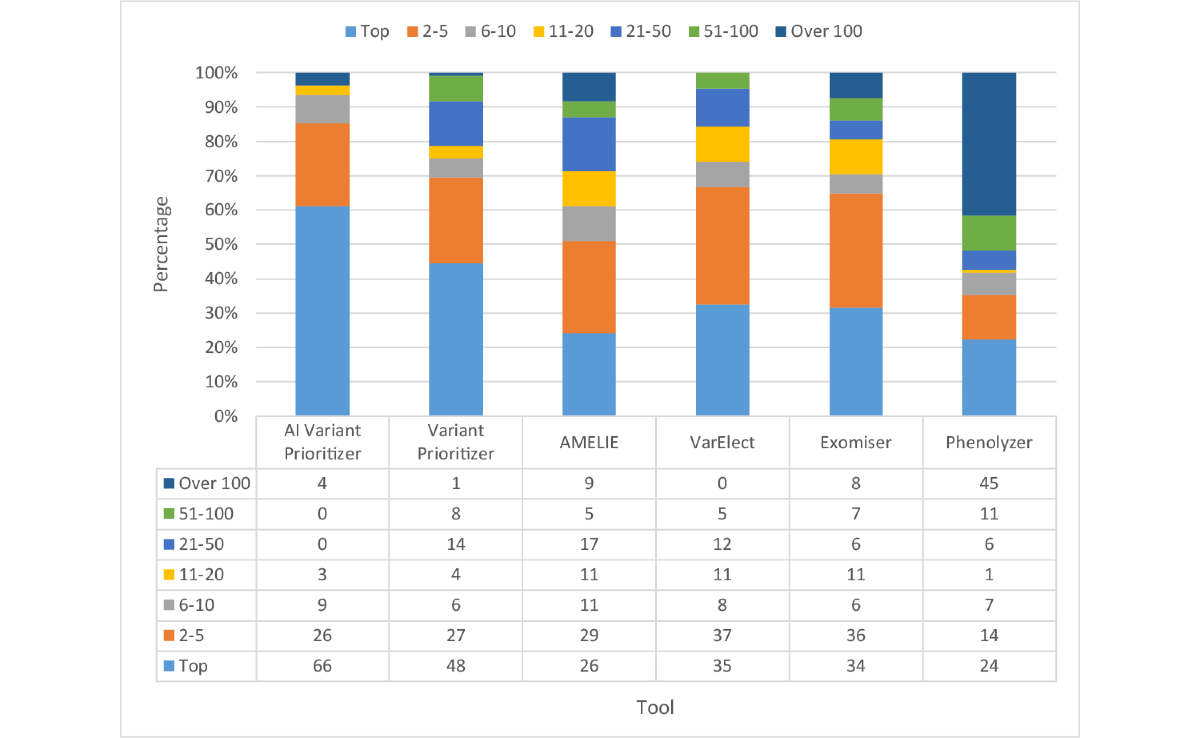
Percentage distribution of ranks. AI: artificial intelligence.

**Figure 8:**
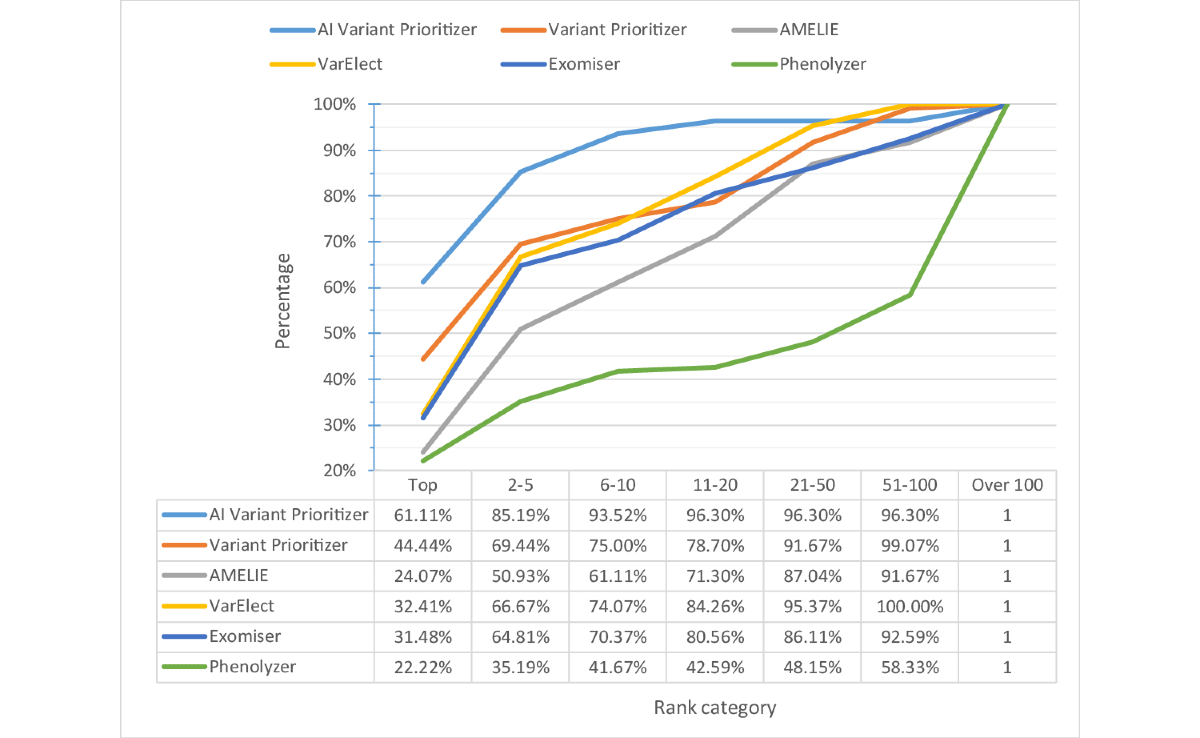
Cumulative percentage distribution of ranks. AI: artificial intelligence.

Figures 7 and 8 show the percentage and cumulative percentage distribution of the target gene ranking for each tool, respectively. From Figure 8, we can see that AI Variant Prioritizer is able to assign the target gene to the top rank for 61.1% (66/108) of the patients, followed by Variant Prioritizer (48/108, 44.4%). It also shows the cumulative rank result, which reveals that our AI Variant Prioritizer has the highest accuracy at ranks 1, 5, 10, and 20. Further, AI Variant Prioritizer shows better performance than other tools. After adopting the HPO terms by looking up the databases, the top 10 ranking list can be increased to 93.5% (101/108).

In comparison with extraction of phenotypic features from SNOMED through manual mapping of HPO terms to SNOMED Clinical Terms (SNOMED CT) [63], our automation approach explores various phenotypic feature extraction tools and focuses on rare disease interpretation. We have also looked into several AI-driven variant prioritization approaches published in the last 3 years, including Fabric GEM [12], MOON [2], and Exomiser. There are several differences between our approach and each of these approaches, including the algorithms used to build the prioritization model, the features considered, and databases integrated. However, the major difference of our approach from others is the method used to turn the relationships between genes and phenotypes into numerical values, which makes way for later prediction. Fabric GEM and MOON utilize Phevor [15] to turn phenotype-gene relationship into numerical values, whereas Exomiser uses PhenoDigm [64] to achieve this goal.

Both Phevor and PhenoDigm construct new methods that bridge HPO and other ontologies to discover more gene-disease associations. Phevor gathers all correlation of diseases and genes provided by HPO and Gene Ontology (GO) and constructs several networks (graphs) and distributes decreasing weights along the paths found. The sum of weights on the specific gene node represents the correlation score of the gene with the corresponding HPO term. PhenoDigm utilizes OWLSim [65] to calculate the similarity among different phenotypes in different ontologies and uses similarity to estimate the magnitude of correlation of given HPO terms and different genes. By contrast, Variant Prioritizer used in our approach extracts the phenotype-gene relationship from a different kind of knowledge source: free text of database. We make a simple comparison of these approaches in Tables 3 and 4.

**Table 3:** The comparison among AI Variant Prioritizer, Fabric GEM, MOON, and Exomiser.

Tool	AI^a^ Variant Prioritizer	Fabric GEM	MOON	Exomiser
Variant scoring algorithm	Random forest	Bayes factor	Decision trees, Bayesian models, neural networks	Rule based
Phenotype-gene score	Variant Prioritizer	Phevor	Phevor	PhenoDigm
Phenotype input format	Plain texts	HPO^b^ terms	HPO terms extracted from electronic health record	HPO terms

^a^AI: artificial intelligence.

^b^HPO: Human Phenotype Ontology.

**Table 4:** The comparison among Variant Prioritizer, Phevor, and PhenoDigm.

Tool	Variant Prioritizer	Phevor	PhenoDigm
Algorithm	Okapi BM25	Graph algorithm	OWLSim
Phenotype input format	Plain texts	HPO^a^ terms	HPO terms
Knowledge base	OMIM^b^, GeneReviews, Entrez Gene and PubTator	HPO and GO^c^	OMIM (HPO), Sanger-MGP [66], MGD [67], and ZFIN [68]

^a^HPO: Human Phenotype Ontology.

^b^OMIM: Online Mendelian Inheritance in Man.

^c^GO: Gene Ontology.

### Feature Importance

**Figure 9:**
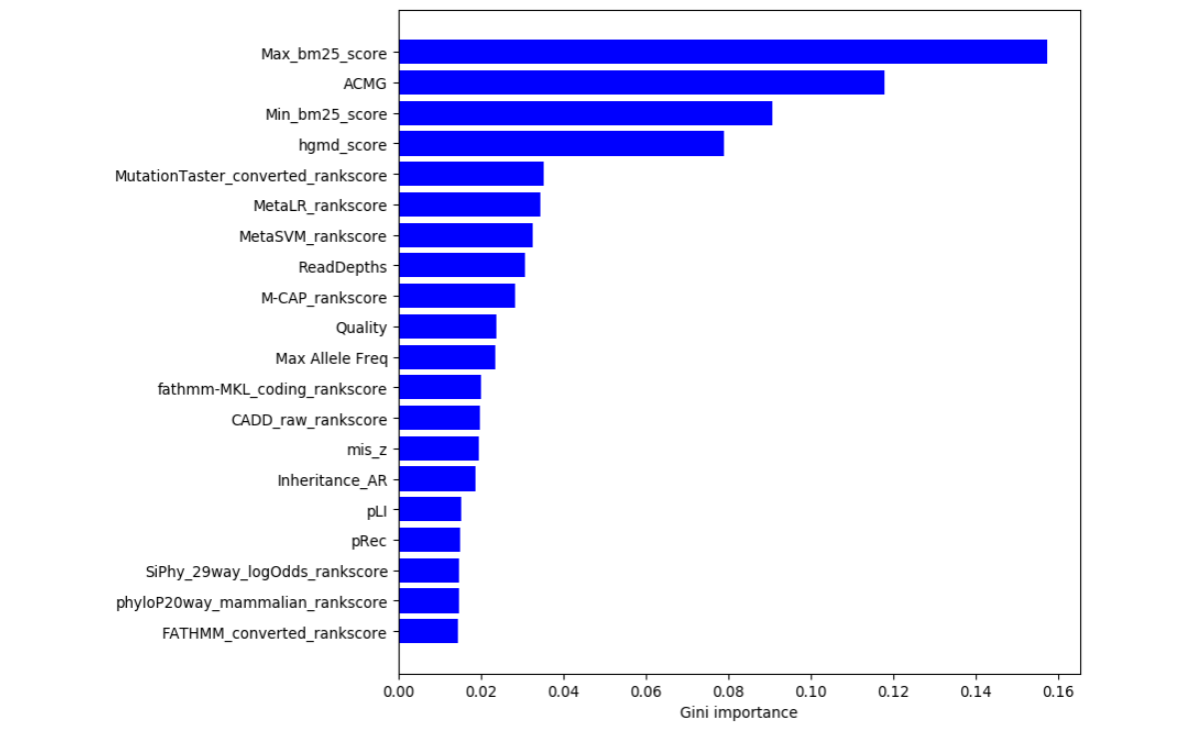
Feature importance.

For interpreting model predictions, we used the feature importance method provided by scikit-learn to identify which feature has the most predictive power. Figure 9 shows the top 20 important features. According to clinical experience, the connection between a variant and phenotype of a patient is a key factor that influences the physician to decide whether to report a variant or not. Similarly, from the figure we can see that the most important feature is the max bm25 score, which refers to the similarity score between the given variant and keywords. Another important factor that influences the reporting decision during clinical analysis is the severity of a variant. The corresponding feature we use is the ACMG score, which is in the second place of feature importance. By contrast, the result of feature importance might provide information for physicians or researchers about the features that they can consider when finding causative variant.

### External Validation

**Figure 10:**
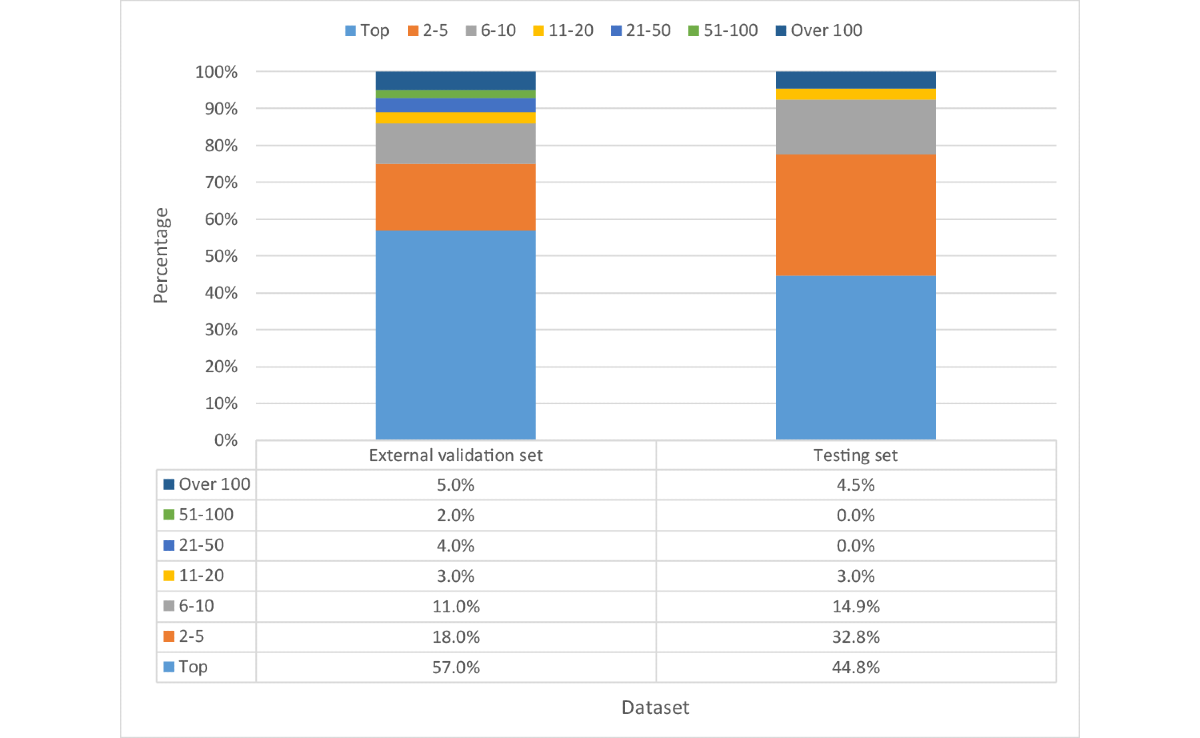
Percentage distribution of ranks.

**Figure 11:**
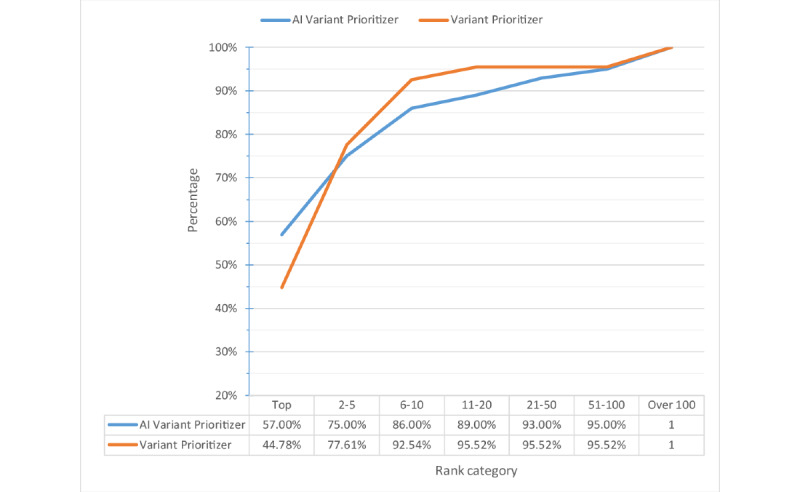
Cumulative percentage distribution of ranks.

We compared the cumulative percentage distribution of ranks from the testing set and the external validation set. The result is shown in Figures 10 and 11. Their percentage values are close to each other in different regions such as top 10 and top 5. The percentage of top 1 rank of the external validation set is even higher than that of the testing set. With this result, we believe that our approach has shown its potential for robust clinical usage.

### Limitations

The study has several potential limitations. First, we could not find massive data for training and testing. This not only restricts the amount of teaching material for the machine learning model, but also restricts available measurements to evaluate the trained model. Second, the gene-phenotype score used in this study did not have enough power to detect small or moderate associations because it relies on how frequently the gene-phenotype relationship is reported to the databases it utilizes. Finally, the study did not adjust for potential confounders, such as diet and physical activity. This could cause potential bias because the way in which genes are expressed can be impacted by lifestyle of patients.

Overall, this study could have potential bias resulting from the lack of sufficient data, lack of reported gene-phenotype relationship, and lack of observation of lifestyle. The impact from the first and the second can be reduced if there are more data and reports available in the future. On the other side, the influence of lifestyle and environment remains an issue that needs more dedicated studies.

### Conclusions

In this research, we proposed a machine learning model, AI Variant Prioritizer, to predict whether a variant is disease causing for patients with rare Mendelian disorder. We have successfully applied sequencing data from WES and free-text phenotypic information of patient’s disease automatically extracted by keyword extraction tools for model training and testing. By interpreting our model, we identified which features of variants are important. Besides, we achieved a satisfactory result on finding the target variant in our testing data set. After testing 108 patients’ WES data, we succeeded in 93.5% (n=101) of the cases to locate the causative variant in the top 10 ranking list among an average of 741 candidate variants per person after the filtering process. The performance of the model is similar to that of manual analysis by the physicians in the Department of Medical Genetics, NTUH, and it has been used to help NTUH with a genetic diagnosis.

As the physicians are very busy almost all the time in taking care of their patients, the search time spent for an accurate genetic diagnosis is extremely important. Our AI predicting model can provide the top 10 hit list with a high probability of 93.5% (101/108), thus helping them save weeks of time if they have to do it manually to search beyond the top 10 list very often.

It is not an easy work to fully interpret the causative variations of a genetic disease. As the precision of the keywords extracted by tools influence the performance of our model, for the future work, we will adopt some NLP techniques such as Bidirectional Encoder Representations from Transformers (BERT) to extract keywords more properly. In addition, the AI Variant Prioritizer model has been built to analyze SNVs and small indels from WES data, but we have not dealt with copy number variations (CNVs) yet. CNVs have been recognized as critical genetic variations, which are associated with both common and complex diseases, and thus have a large influence on several Mendelian and somatic genetic disorders. Therefore, we will collect data on CNVs and extend the capability of our system to annotate and filter CNVs. Furthermore, we will enlarge our data set by adding CNVs as our training data to enable the AI Variant Prioritizer model to predict any kind of causative genetic variations. Before implementation of AI Variant Prioritizer, the mean turnaround time of the entire WES pipeline, from DNA extraction to clinical diagnosis, was 5.8 (SD 1.1) days using Variant Prioritizer. However, after implementation of AI Variant Prioritizer, the mean turnaround time was reduced to 4.8 (SD 1.2) days for rapid trio exome sequencing analysis in NTUH.

## Appendix

Multimedia Appendix 1 Allele frequency.

Multimedia Appendix 2 Description of features used in this research.

Multimedia Appendix 3 Target variants, HPO term, and keywords of test case. HPO: Human Phenotype Ontology.

## Abbreviations

ACMG: American College of Medical Genetics and Genomics
AI: artificial intelligence
AMP: Association for Molecular Pathology
API: application programming interface
BERT: Bidirectional Encoder Representations from Transformers
CNV: copy number variation
EMR: electronic medical record
GBDT: gradient boosting decision tree
gnomAD: Genome Aggregation Database
GO: Gene Ontology
HGMD: Human Genome Mutation Database
HIPAA: Health Insurance Portability and Accountability Act
HPO: Human Phenotype Ontology
IRB: institutional review board
MViewer: Mutation Viewer
NGS: next-generation sequencing
NLP: natural language processing
NTUH: National Taiwan University Hospital
OMIM: Online Mendelian Inheritance in Man
SNV: single-nucleotide variant
SVM: support vector machine
UMLS: Unified Medical Language System
VCF: variant call format
VEP: Variant Effect Predictor
