# Supplementary material for: Diagnosis of a Single-Nucleotide Variant in Whole-Exome Sequencing Data for Patients With Inherited Diseases: Machine Learning Study Using Artificial Intelligence Variant Prioritization
Source: JMIR Bioinform Biotechnol. 2022 Sep 15;3(1):e37701. doi: 10.2196/37701 (PMC11168239; doi:10.2196/37701)
Supplement: Multimedia Appendix 1 [file bioinform_v3i1e37701_app1.docx]

## Multimedia Appendix 1

Allele Frequency

After variant annotation by ANNOVAR, we get allele frequencies from 1,000 Genomes Project, ExAC, ESP, and gnomAD database. Since most of our patients are Taiwanese, we also import allele frequency of variants from Taiwan Biobank, a genomic database of the Taiwanese population. Instead of using all of these columns, we define a new column called max minor allele frequency. The value of max minor allele frequency column is the maximum value of all these minor allele frequency columns.
